# Iron-Ascorbate-Mediated Lipid Peroxidation Causes Epigenetic Changes in the Antioxidant Defense in Intestinal Epithelial Cells: Impact on Inflammation

**DOI:** 10.1371/journal.pone.0063456

**Published:** 2013-05-22

**Authors:** Sabrina Yara, Jean-Claude Lavoie, Jean-François Beaulieu, Edgard Delvin, Devendra Amre, Valerie Marcil, Ernest Seidman, Emile Levy

**Affiliations:** 1 Department of Nutrition, Research Centre, CHU-Sainte-Justine, Université de Montréal, Montreal, Quebec, Canada; 2 Department of Pediatrics, Research Centre, CHU-Sainte-Justine, Université de Montréal, Montreal, Quebec, Canada; 3 Canadian Institutes for Health Research Team on the Digestive Epithelium, Department of Anatomy and Cellular Biology, Faculty of Medicine and Health Sciences, Université de Sherbrooke, Sherbrooke, Quebec, Canada; 4 Department of Biochemistry, Research Centre, CHU-Sainte-Justine, Université de Montréal, Montreal, Quebec, Canada; 5 Research Institute, McGill University, Campus MGH, C10.148.6, Montreal, Quebec, Canada; North Carolina State University, United States of America

## Abstract

**Introduction:**

The gastrointestinal tract is frequently exposed to noxious stimuli that may cause oxidative stress, inflammation and injury. Intraluminal pro-oxidants from ingested nutrients especially iron salts and ascorbic acid frequently consumed together, can lead to catalytic formation of oxygen-derived free radicals that ultimately overwhelm the cellular antioxidant defense and lead to cell damage.

**Hypothesis:**

Since the mechanisms remain sketchy, efforts have been exerted to evaluate the role of epigenetics in modulating components of endogenous enzymatic antioxidants in the intestine. To this end, Caco-2/15 cells were exposed to the iron-ascorbate oxygen radical-generating system.

**Results:**

Fe/Asc induced a significant increase in lipid peroxidation as reflected by the elevated formation of malondialdehyde along with the alteration of antioxidant defense as evidenced by raised superoxide dismutase 2 (SOD2) and diminished glutathione peroxidase (GPx) activities and genes. Consequently, there was an up-regulation of inflammatory processes illustrated by the activation of NF-κB transcription factor, the higher production of interleukin-6 and cycloxygenase-2 as well as the decrease of IκB. Assessment of promoter’s methylation revealed decreased levels for SOD2 and increased degree for GPx2. On the other hand, pre-incubation of Caco-2/15 cells with 5-Aza-2′-deoxycytidine, a demethylating agent, or Trolox antioxidant normalized the activities of SOD2 and GPx, reduced lipid peroxidation and prevented inflammation.

**Conclusion:**

Redox and inflammatory modifications in response to Fe/Asc -mediated lipid peroxidation may implicate epigenetic methylation.

## Introduction

Reactive Oxygen Species (ROS) are by-products of normal aerobic metabolism. Various studies have evidenced their effectiveness as important signaling molecules that modulate gene expression, cell growth and survival, as well as oxygen sensing in various cell types [1], [2]. However, their excessive formation leads to lasting oxidative stress (OxS), characterized by an imbalance between oxidant-producing systems and antioxidant defense mechanisms, which can trigger cell damage by oxidizing macromolecular structures (lipids, proteins and DNA) and causes cell death [3]. Thus, depending on their cell concentrations, ROS can act as either beneficial or harmful biological agents that contribute to the development of chronic diseases, including osteoporosis, type 2 diabetes, neurodegenerative, cardiovascular disorders, and cancer [4].

Normally, cells struggle to efficiently remove ROS in order to avoid deleterious effects. To this end, several enzymes metabolize reactive species and their byproducts, thereby reducing OxS. For example, the superoxide dismutase enzyme (SOD) acts as an endogenous cellular defense system that converts superoxide anion into oxygen and hydrogen peroxide, with the latter being further detoxified by catalase and glutathione peroxidase (GPx).Variation in their genes may impact their enzymatic antioxidant activity and, thus, the ability to scavenge, neutralize and remove reactive species, to inhibit oxidative chain reactions, chelate reactive metals and repair damage to biological molecules. For example, single nucleotide polymorphisms in genes that code for endogenous antioxidant enzymes or proteins involved in dietary antioxidant uptake and utilization may have a direct impact on the ability to manage OxS and prevent subsequent disease development in humans [5]–[7]. Individual genetic variation may also influence dietary antioxidant status in a fashion of gene-diet interactions and, consequently, the body’s ability to manage OxS [8].

Recent data in the turning on/off of genes and gene regulation in organism proteins have converged with the discoveries of epigenetic mechanisms. In fact, epigenetic modifications of DNA alter gene expression profiles, and phenotypes as well as portions of the DNA that are transcribed can be turned on or off depending on the epigenetic modifications physically acting at specific loci of the genome. It has already been demonstrated that environmental exposures, such as diets, tobacco and alcohol use, physical activity, OxS and exposure to chemical carcinogens can influence the epigenome [9]–[11]. Although the information is still very limited, it seems that the endogenous antioxidants are not immune to this type of regulation. Notably, SOD2 transcriptional activity may be controlled at least in part via epigenetic mechanisms at different stages in the development of human cancer by processes that include histone methylation, histone acetylation and DNA methylation [12], [13]. Similarly, hypermethylation of GPx promoter is frequently observed in a wide spectrum of human malignancies [14].

Growing attention is being paid to the implication of OxS in various intestinal disorders. Marked increase of ROS may contribute to disruption of the intestinal epithelial barrier and the subsequent development of systemic inflammation by enabling influx of endotoxin and other noxious luminal contents into the systemic circulation as it is the case for chronic kidney disease [15], cystic fibrosis [16] and inflammatory bowel diseases [17]. Mucosal antioxidant defense is altered as very often evidenced by attenuated redox status that contributes to disease progression and exacerbation of the pathological states [18]. Accordingly, obesity- and diabetes-associated inflammation and intestinal lipoprotein overproduction may be related to local OxS involving weak antioxidant defense [19], [20]. Under all these pathophysiological conditions, a substantial lowering was recorded in endogenous antioxidants, including SODs and GPx [16], [21]–[23]. In light of these numerous findings, understanding the regulation of intestinal endogenous enzymatic antioxidants is of the utmost importance since they provide solid protection against stress-induced diseases. In particular, it has not been established whether OxS alters the antioxidant status by acting on the epigenetic machinery, more specifically the DNA-methylation pathway. To tackle this issue, we evaluated how the iron-ascorbate (Fe/Asc) oxygen radical-generating system modulated SOD2 and GPx enzymes in the Caco-2/15 cell line, a reliable human intestinal model. We examined whether regulatory changes in SOD2 and GPx2 expression are attributable to epigenetic mechanisms. Evidence for the contribution of methylation of cytosines within CpG sites in their DNA was tested by measuring their activities and by using the demethylation agent 5-Aza-2′-deoxycytidine (5-AZA).

## Materials and Methods

### Cell Culture and Treatments

The human epithelial colorectal adenocarcinoma Caco-2/15 cell line, a stable clone of the parent Caco-2 cells (American Type Culture Collection, Rockville, MD), was obtained from Dr. JF Beaulieu (Department of Cellular Biology, Faculty of Medicine, Université de Sherbrooke, Sherbrooke, Quebec, Canada). Caco-2/15 cells were grown at 37°C with 5% CO_2_ in minimal essential medium (MEM) (GIBCO-BRL, Grand Island, NY) containing 1% penicillin-streptomycin, 1% MEM nonessential amino acids (GIBCO-BRL) and supplemented with 10% decomplemented fetal bovine serum (FBS) (Flow, McLean, VA) as described previously [24]–[27]. Caco-2/15 cells (passages 20–25) were maintained in T-75 cm2 flasks (Corning Glass Works, Corning, NY) and were split (1∶5) when they reached 80–90% confluence by using 0.05% trypsin-0.5 mM EDTA (GIBCO-BRL). For individual experiments, cells were plated at a density of 5×10^5^ cells/well on 6-well plate with flat bottom (Costar, Cambridge, MA), in MEM containing 10% FBS and grown for 2 days. Before exposure to Fe/Asc (200 µM/2 mM) for 6 h at 37°C, Caco-2/15 cells were pre-incubated with 0.25 mM 6-hydroxy-2,5,7,8-tetramethylchromane-2-carboxylic acid (Trolox), with ethanol as a vehicle, for 24 h. Four experimental cell groups were considered: control (with 0.25 mM ethanol as a vehicle), Fe/Asc (to induce OxS), Trolox (as antioxidant) and Trolox+Fe/Asc (to assess the direct contribution of OxS via its neutralisation by the antioxidant). For the studies of DNA methylation, Caco-2/15 cells were pre-incubated with 5-AZA at a concentration of 10 µM [28] for 54 h [28], [29], and 5 experimental cell groups were established: control (with ethanol as a vehicle), 5-AZA (to preclude methylation), Fe/Asc+5-AZA (to prevent methylation in the presence of OxS), Trolox+5-AZA (to evaluate the combined effect of antioxidant and demethylating agent), and Fe/Asc+Trolox+5-AZA (to evaluate the combined effect of antioxidant and demethylating agent in the presence of OxS).

### Cellular Viability Assays

Cellular proliferation and viability were evaluated using the 3-(4,5-dimethyldiazol-2-yl)-2,5 diphenyl Tetrazolium Bromide (MTT, Sigma) assay. At the end of cell incubation, the medium was aspirated and replaced with an MTT solution (0.5 mg/mL) and cells were incubated for 2 h at 37°C with 5% CO_2_ to allow MTT oxidation by the succinate dehydrogenase enzyme in viable cells. The MTT solution was then aspirated and 500 µl of dimethyl sulfoxide was added to each well to dissolve the resulting blue formazan crystals. The absorbance was measured at 535 nm with DMSO as a blank.

### Lipid Peroxidation Markers

Lipid peroxidation was estimated by measuring the release of free malondialdehyde (MDA) from Caco-2/15 cells in the culture medium by HPLC as described previously [24]–[26]. Proteins were first precipitated with a 10% sodium tungstate solution (Sigma). The protein-free supernatants then reacted with an equivalent volume of 0.5% (wt/v) thiobarbituric acid solution (Sigma) at 95°C for 60 min. After cooling to room temperature, the pink chromogene [(thiobarbituric acid) 2-MDA] was extracted with 1-butanol and dried over a stream of nitrogen at 37°C. The dry extract was then resuspended in a potassium dihydrogen phosphate-methanol mobile phase (70∶30, pH 7.0) before MDA determination by HPLC with fluorescence detection.

### Western Blotting

To determine the protein expression of cyclooxygenase-2 (COX-2), IκB, nuclear factor-κB (NF-κB) and interleukin (IL)-6, Caco-2/15 cells were collected in mammalian protein extraction reagent (Thermo Fisher Scientific, Rockford, IL) containing a mixture of antiproteases (Roche Diagnostics, Laval, QC). Cells were sonicated (five burst) and centrifuged at 15,000×*g* for five minutes at 4°C and the supernatant was transferred to a fresh tube. The protein concentration of each sample was determined by Bradford assay (Bio-Rad, Mississauga, ON). Proteins (30 µg) were denatured at 95°C for 5 min in sodium dodecyl sulfate (SDS), dithiothreitol and β-mercaptoethanol-containing sample buffer, separated on a 12% gradient SDS/PAGE, and electroblotted onto Hybond-C Extra nitrocellulose membranes (Amersham) in 192 mM glycine and 25 mM Tris-base. Membranes were then blocked for 60 min at room temperature with solution containing 50 mM Tris-base, 150 mM sodium chloride (NaCl), 0.1% Tween and 5% non-fat dry milk. The different primary antibodies were added as follow: 1∶1000 rabbit anti-COX-2 (74 kDa; Novus, Oakville, ON), 1∶5000 goat anti-NF-κB (65 kDa; Santa Cruz Biotechnology, Santa Cruz, CA), 1∶5000 rabbit anti-IκBα (39 kDa; Cell signaling, Beverly, MA) and 1∶500 mouse anti-IL-6 (25 kDa; R&D Systems, Minneapolis, MN). After overnight incubation, species-specific horseradish peroxidase-conjugated secondary antibodies (Jackson Laboratory, Bar Harbor, Maine) were added for 1 h at room temperature to detect the primary antibodies. The β-actin protein expression was determined to confirm equal loading (Fermentas, Glen Burie, Maryland). Protein mass was quantified using an HP Scanjet scanner equipped with a transparency adapter and the UN-SCAN-IT gel 6.1 software.

### Enzymatic Activity of Endogenous Antioxidant Enzymes

The activities of SOD2, GPx and G-Red was measured as described previously [26]. Briefly, Caco-2/15 cells were harvested in hypotonic lysis buffer (10 mM HEPES, 1.5 mM MgCl_2_, 10 mM KCl, 0.5 mM DTT, and 0.2 mM PMSF). Total SOD2 activity was determined as described by McCord et al [30]. Briefly, superoxide radicals (O_2_^−^) were generated by the addition of xanthine and xanthine oxidase, and oxidation of the SOD assay cocktail was followed using a spectrophotometer at 550 nm for 5 min. The same reaction was repeated with addition of the sample, and the SOD assay cocktail was less oxidized because of the SOD activity in the sample. Total SOD activity was then calculated in the presence or absence of potassium cyanide (1 mM) that allows defining the contribution of MnSOD activity.

For GPx activity, aliquots of cell homogenates were added to a PBS buffer containing 10 mM GSH, 0.1 U of G-Red, and 2 mM NADPH with 1.5% H_2_O_2_ to initiate the reaction. Absorbance was monitored every 30 s at 340 nm for 5 min.

For G-Red activity, cell homogenates were added to a PBS buffer containing 2 mM NADPH and 10 mM GSSG to initiate the reaction. Absorbance was monitored every 30 s at 340 nm for 5 min.

### RNA Isolation and Reverse Transcription

Total RNA was extracted from Caco-2/15 cells using TRIzol reagent (Invitrogen), and its amount was determined by spectrophotometer. Moloney murine leukemia virus reverse transcriptase (Invitrogen) was used to obtain cDNA according to the manufacturer’s instructions. Reverse transcription lasted 50 min at 37°C, and a quantity of 1 µg total RNA was added in each reaction.

### Determination of mRNA by PCR

cDNA was amplified by PCR using a *Taq* polymerase (Feldan Bio, Quebec, QC, Canada) according to the manufacturer’s instructions. Briefly, 20–28 cycles of amplification were used at 95°C for 30 s, annealing temperature for 30 s, and 72°C for 30 s. The primers used for the SOD2 amplification were: sense primer 5′-CGACCTGCCCTACGACTACG-3′ and antisense primer 5′-TGACCACCACCATTGAACTT-3′. Annealing temperature was 58°C and 22 cycles were used to obtain amplicon (198 bp). The primers used for the GPx amplification were: 5′-TTCGCTCTGAGGCACAACC-3′ and antisense primer 5′-ACAGGGCTCCAAATGATGAG-3′. Annealing temperature was 58°C and 28 cycles were used to obtain amplicon (151 bp). PCR was performed using the UNO II thermocycler (Biometra). Amplicons were visualized on standard ethidium bromide-stained agarose gels. The number of amplification cycles corresponds to the linear portion of the exponential phase, as determined in preliminary experiments. Fold induction was calculated using GAPDH as a housekeeping gene, and quantification was determined with the software UN-SCAN-IT gel 6.1.

### Genomic DNA Isolation and Bisulfite Modification

DNA was isolated and modified (unmethylated cytosines) employing the EZ DNA Methylation-Direct Kit (Zymo Research, Irvine, CA) according to the manufacturer’s instructions.

### Polymerase Chain Reaction of Bisulfite-treated DNA

Two sequential PCRs were performed to amplify the modified DNA fragments of interest [31], [32]. 1 µl of modified DNA was used in the first PCR (a) and 1 µl of PCR product was used in the subsequent PCR (b). The primers used for the SOD2 amplification were: sense primer 5′-GTA TTT TTA GGG G[C/T]G GAT [C/T]GG AGG TAG GGT TT-3′ and antisense primer 5′-CCA AAC CC[A/G] ATA C[A/G]A CCA CTA TC[A/G] CCA TTA C-3′, sense primer 5′-GGG T[C/T]G TAT TAA TTT TA[C/T] GGG GGT AGG GGT-3′ and antisense primer 5′-AAC CCC TTA CCC CTT AAA AC[A/G] TAA CC[A/G] AAT CCC-3′.

Conditions for the first PCR (a) were: initial enzyme activation at 95°C for 5 min followed by 35 cycles at 94°C 1 min for denaturation, annealing at 55°C for 1 min and extension at 72°C for 1 min, and final extension at 72°C for 10 min. For the second PCR (b), the same conditions were used. The PCR reaction of 50 µl contained 10×CoralLoad PCR buffer (Qiagen, Toronto, Ontario, Canada), 25 mM MgCl_2_, 200 µM of each dNTP, 0.5 µM of each primer and 2.5 units HotStarTaq DNA polymerase (Qiagen, Toronto, Ontario, Canada).

A volume of 1 µl of modified DNA and the following primers were used for GPx2 promoter amplification.

Sense primer 5′-GAG AAG AA[C/T] GTG AAT AGG AAT PGX2-3′ and antisense primer 5′-CA ATA AAA ACC ATA ATA AAA C[A/G]C-3′.

PCR conditions and reactions were the same as those described above except the annealing temperature that was 54°C.

### Statistical Analysis

All values are expressed as the mean ± SEM. The data were analysed by ANOVA with Prism 5.01 (GraphPad Software) and the differences between the means were assessed post-hoc using Tukey’s test.

## Results

### Cellular Viability Following Various Treatments

**Figure 1:**
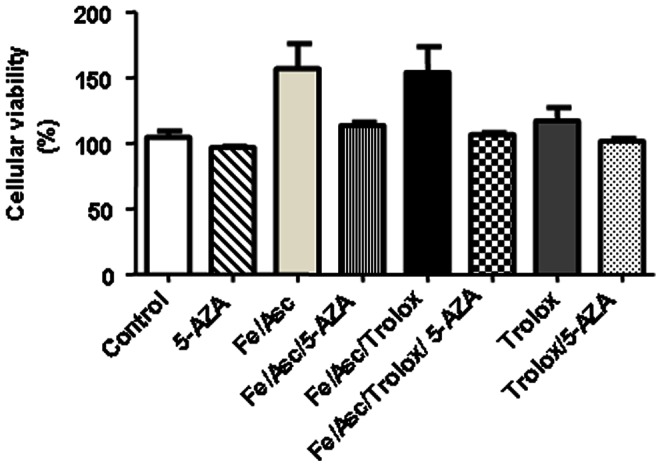
Effects of iron/ascorbate and 5-Aza-2′-deoxycytidine on cell viability in Caco-2/15 cells. Caco-2/15 cells were incubated with Fe/Asc (200 µM/2 mM) and Trolox (0.25 mM) for 6 h at 37°C and/or with 5-AZA (10 µM) as described in Materials and Methods, Cellular viability was assessed by MTT. Results represent the means ± SEM of n = 3 independent experiments.

Caco-2/15 cell viability was not affected by the addition of the various treatments. Therefore, it could be concluded that our experimental conditions, including the use of Fe/Asc, 5′-AZA or combination of both, did not exert any cytotoxic effects on Caco-2/15 cells following a 6 h-incubation (Figure 1).

### Malondialdehyde Production Following Fe/Asc Treatment

**Figure 2:**
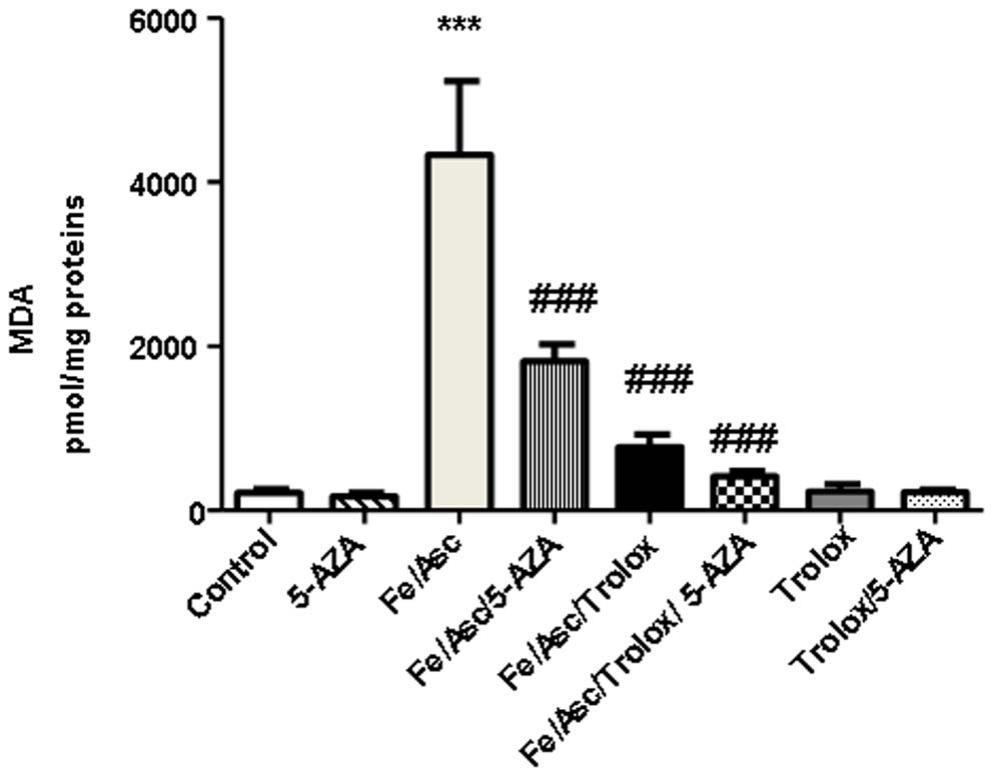
Malondialdehyde concentrations in Caco-2/15 cells challenged with iron/ascorbate and/or Trolox. Caco-2/15 cells were exposed to Fe/Asc (200 µM/2 mM) and Trolox (0.25 mM) for 6 h at 37°C and/or with 5-AZA (10 µM). Oxidative stress was assessed by measuring MDA as an index of lipid peroxidation. Values are means ± SEM for three independent experiments. ***P<0.001 vs. controls; ^###^P<0.001 vs. Fe/Asc.

Before evaluating the impact of OxS on various cellular processes, we assessed the effectiveness of Fe/Asc in initiating lipid peroxidation after incubation with Caco-2/15 cells. At the end of a 6 h-culture period, the degree of lipid peroxidation was determined by measuring MDA in cells. As illustrated in Figure 2, Fe/Asc induced a significant increase in MDA levels above baseline values compared with control cells. The concentration of MDA was 20-fold higher in cells supplemented with Fe/Asc compared to untreated cells. Pre-incubation with the strong antioxidant Trolox markedly limited the production of MDA and displayed the ability to maintain MDA concentration near baseline values, providing direct evidence for the ability of the Fe/Asc system to provoke profound lipid peroxidation. Interestingly, the combination of Trolox and 5-AZA was more effective in reducing MDA levels (Figure 2).

### Inflammation Induction in Response to Fe/Asc Exposition

**Figure 3:**
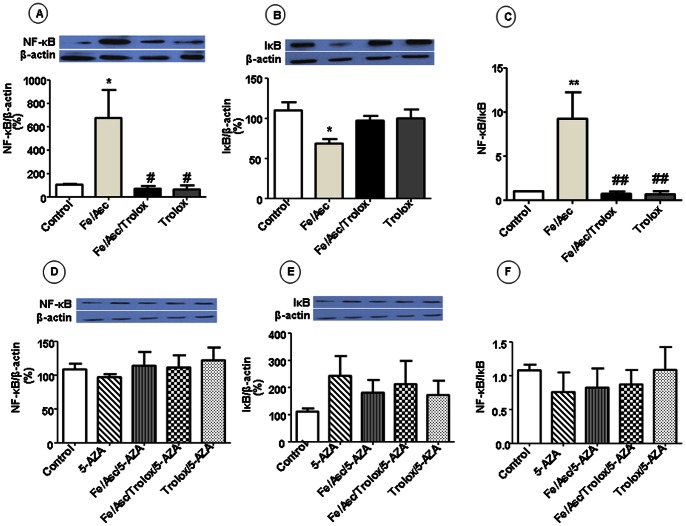
Effects of oxidative stress on transcription factor NF-κB in Caco-2/15 cells. Caco-2/15 cells were incubated with Fe/Asc (200 µM/2 mM) and Trolox (0.25 mM) for 6 h at 37°C and/or with 5-AZA (10 µM). The protein expression of NF-κB (A, D) and IκB (B, E) were determined by western blot as described in Materials and Methods. Then the NF-κB/IκB was calculated (C, F). Results represent the means ± SEM of n = 3 independent experiments. *P<0.05, **P<0.01 vs. controls; ^#^P<0.05, ^##^P<0.01 vs. Fe/Asc.

**Figure 4:**
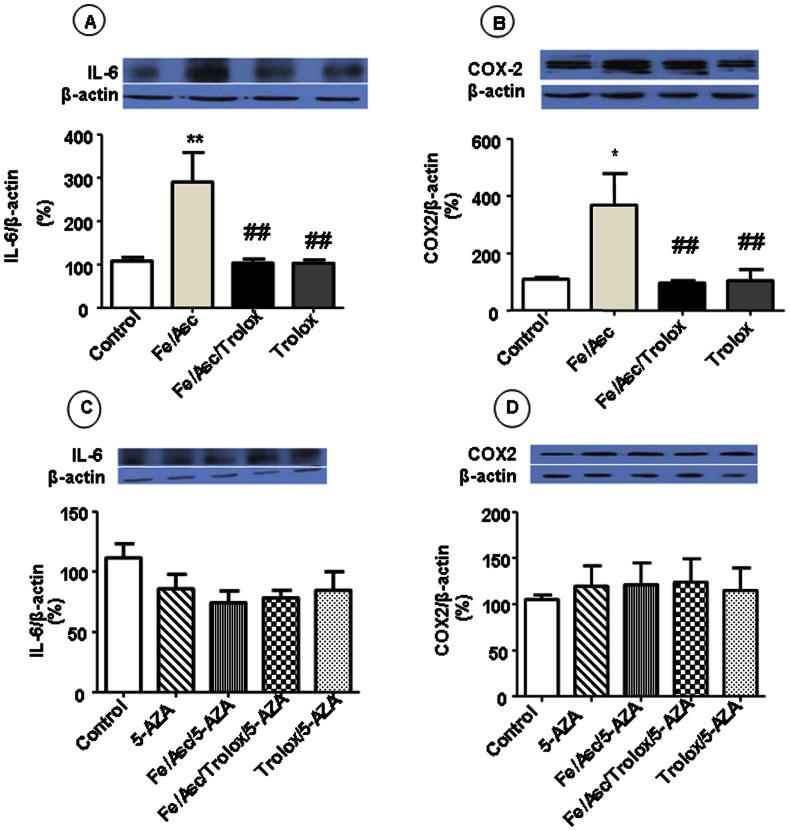
Effects of oxidative stress on inflammatory markers in Caco-2/15 cells. Cells were incubated with Fe/Asc (200 µM/2 mM) and Trolox (0.25 mM) for 6 h at 37°C and/or with 5-AZA (10 µM). The protein expression of Interleukin-6 (IL-6) (A, C) and cyclooxygenase 2 (COX2) (B, D) was determined by western blot as described in Materials and Methods. Results represent the means ± SEM of n = 3 independent experiments. *P<0.05, **P<0.01 vs. controls; ^#^P<0.05, ^##^P<0.01 vs. Fe/Asc.

Since inflammation and OxS are closely associated, we examined the protein expression of NF-κB, a powerful transcription factor endowed with the high capacity to activate the inflammatory pathway. We also assessed the protein expression of IκB that prevents NF-κB translocation to nucleus for transcription activation of pro-inflammatory genes. Western Blot analysis revealed a marked rise of NF-κB protein (Figure 3A) and decrease of IκB (Figure 3B) protein expression following incubation of Caco-2/15 cells with Fe/Asc. Consequently, the NF-κB/IκB protein ratio was higher (Figure 3C **)** in experimental cells in comparison with control cells. Nevertheless, pre-incubation with Trolox restored both of them to control levels. As expected from the NF-κB data, measurement of the cytokine IL-6, a pro-inflammatory component produced by cells in response to injury, revealed the same trend of increase (Figure 4A **)**. We finally noted in Caco-2/15 cells, incubated with Fe/Asc, higher levels of COX-2 (Figure 4B), a highly regulated enzyme that catalyzes the production of prostaglandins under pathologic conditions. COX-2 values returned to normal with the administration of Trolox (Figure 4B **)**. Overall, these results provide direct evidence for the ability of the Fe/Asc system to provoke not only lipid peroxidation, but also inflammation in intestinal epithelial cells.

### Profile of Endogenous Antioxidants Following Fe/Asc Administration

**Figure 5:**
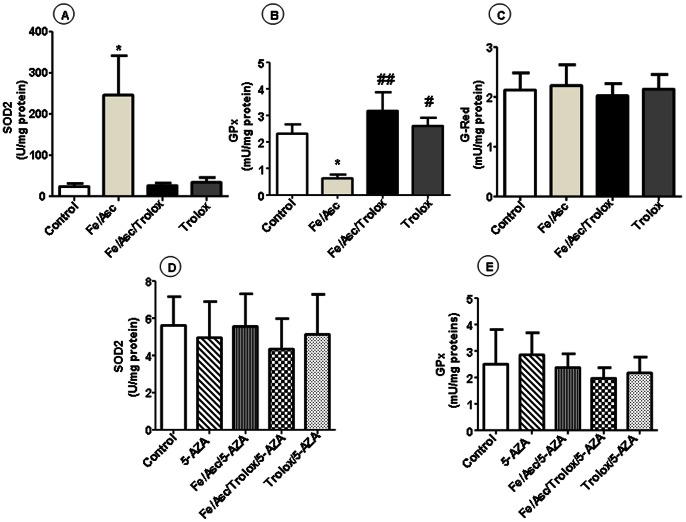
Effects of oxidative stress on regulatory endogenous antioxidant activities in Caco-2/15 cells. Cells were incubated with Fe/Asc (200 µM/2 mM) and Trolox (0.25 mM) for 6 h at 37°C and/or with 5-AZA (10 µM). The activities of SOD2 (A, D), GPx (B, E) and G-Red (C) were then measured as described in Materials and Methods. Results represent the means ± SEM of n = 3 independent experiments. *P<0.05 vs. controls; ^#^P<0.05, ^##^P<0.01 vs. Fe/Asc.

Since cells have developed an enzymatic antioxidant pathway against free radicals and ROS, which are generated during oxidative metabolism, we first measured the activity of SOD2 that converts superoxide anion to hydrogen peroxide. The treatment of Caco-2/15 cells with Fe/Asc led to a significant elevation of the SOD2 activity (Figure 5A). Addition of the antioxidant Trolox cancels the effects of Fe/Asc. The next experiments aimed to assess the anti-oxidative response of GPx, an important enzyme that catalyzes the conversion of hydrogen peroxide to water. The addition of Fe/Asc to Caco-2/15 cells induced a significant decrease in GPx activity (Figure 5B). We finally measured glutathione reductase (GR) that reduces glutathione disulfide to the sulfhydryl form. No significant changes were found when Caco-2/15 cells were treated with Fe/Asc (Figure 5C).

### SOD2 and GPX Gene Expression Following Fe/Asc Exposition

**Figure 6:**
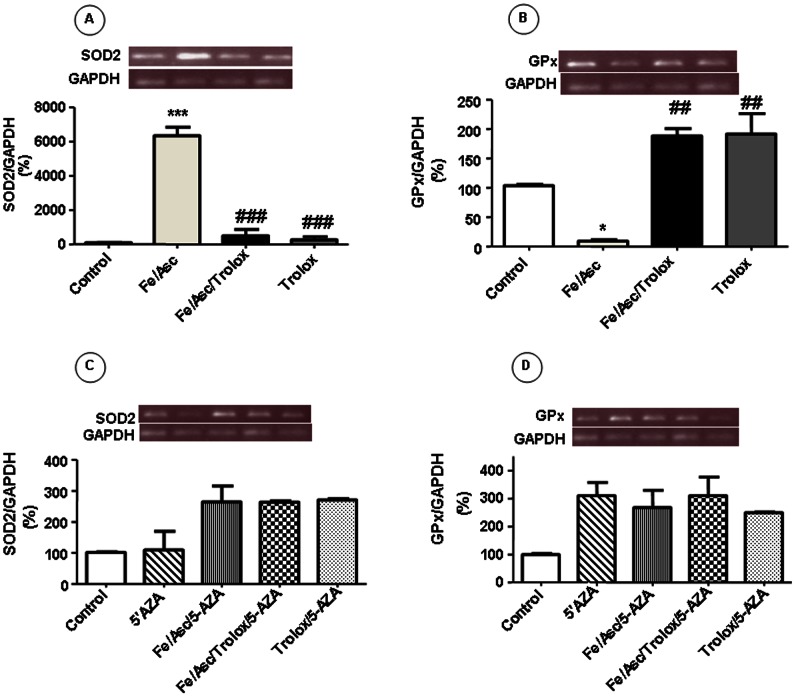
Effects of oxidative stress on antioxidant genes in Caco-2/15 cells. Cells were incubated with Fe/Asc (200 µM/2 mM) and Trolox (0.25 mM) for 6 h at 37°C and/or with 5-AZA (10 µM). Gene expression of SOD2 (A, C) and GPx (B, D) were then measured as described in Materials and Methods. Results represent the means ± SEM of n = 3 independent experiments. *P<0.05, ***P<0.001 vs. controls; ^##^P<0.01, ^###^P<0.001 vs. Fe/Asc.

The mRNA expression of antioxidant genes SOD and GPX was analyzed to determine whether it presented the same trend as the enzymatic activities. The treatment of Caco-2/15 cells with Fe/Asc led to a significant elevation of the SOD2 transcript (Figure 6A) and marked decrease of the GPx transcript (Figure 6B). Addition of the antioxidant Trolox suppressed the modulatory effects of Fe/Asc.

### SOD2 and GPX2 Promoter Methylation Following Fe/Asc Exposition

**Figure 7:**
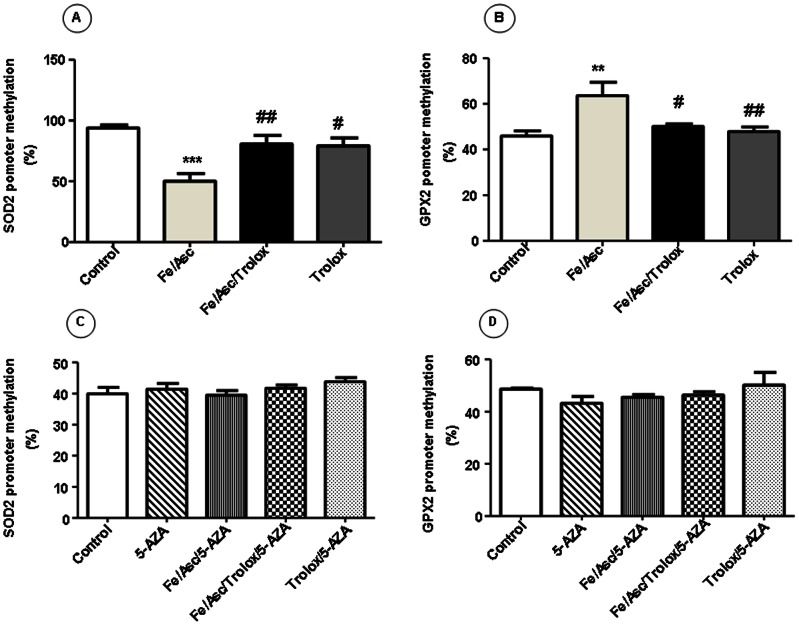
SOD2 and GPX2 promoter methylation following Fe/Asc exposition. Cells were incubated with Fe/Asc (200 µM/2 mM) and Trolox (0.25 mM) for 6 h at 37°C and/or with 5-AZA (10 µM). DNA methylation of SOD2 (A, C) and GPx2 (B, D) was then determined as described in Materials and Methods. Results represent the means ± SEM of n = 3 independent experiments. **P<0.01, ***P<0.001 vs. controls; ^#^P<0.05, ^##^P<0.01 vs. Fe/Asc.

As Fe/Asc induced a rise in SOD2 activity in Caco-2/15 cells, we sought to investigate whether potential mechanisms implicate DNA methylation. Exposure of Caco-2/15 cells to Fe/Asc oxygen radical-generating system decreased methylation of the SOD2 promoter (44% less compared to controls). Pre-incubation with Trolox before the addition of Fe/Asc almost maintained the SOD2 promoter methylation at the level of controls (Figure 7A). Conversely, GPx_2_ promoter was more methylated when Caco-2/15 cells were treated with Fe/Asc (18% more compared to controls). Once again, Trolox prevented the increase in the GPX_2_ promoter methylation caused by the addition of Fe/Asc (Figure 7B). These results demonstrate that Fe/Asc-mediated OxS alters DNA methylation modulation of SOD2 and GPx2 genes in opposite fashion.

### Reactivation of SOD2 and GPx2 Expression after Treatment with 5-Aza-2′-deoxycytidine

We treated Caco-2/15 cells with 5-AZA, a strong demethylating agent to determine whether the activity of SOD2 and GPx2 as well as their DNA methylation, could be reactivated. As shown in Figures 5D and 5E, the activity of both enzymes was re-established at the same level of control cells after treatment with 5-AZA. In parallel, 5-AZA restored gene expression (Figures 6C and 6D) and the DNA methylation (Figures 7C and 7D **)** of these antioxidants and counteracted all the Fe/Asc effects. Moreover, it resulted in the substantial reduction of MDA, but without a complete neutralization of lipid peroxidation (Figure 2), the prevention of NF-κB increase (Figure 3D) and IκB decrease (Figure 3E) protein expression as well as in normalization of NF-κB/IκB ratio (Figure 3F), IL-6 and COX-2 protein expression (Figures 4C and 4D).

## Discussion

Lipid peroxides are a major source of dietary oxidants of mutagenic or carcinogenic potential that are of nutritional and toxicological importance [33], [34]. In addition, the intestine is exposed to intraluminal oxidants from catalase-negative bacteria to oxidase-producing desquamated cells (e.g. xanthine oxidase) that amplify the generation of free radicals, and to hypothiocyanous acid-containing saliva (formed from the interaction between salivary peroxidases with H_2_O_2_ and thiocyanate), which may increase luminal reactive oxygen metabolite content [35], [36]. Clearly, the ingestion and/or occurrence of peroxides may have implications for human health, particularly in the long term since they may cause transient or permanent damage to cellular constituents, including nucleic acids, proteins, lipids, and membranes [37]–[39]. By virtue of their ability to generate oxyradicals, lipid peroxides are able to initiate degenerative processes and promote digestive system disorders, such as inflammation and cancer [40], [41]. The presence of potent cellular detoxification systems minimizes radical generation, terminates radical processes, and repairs damaged macromolecules [42]. Nevertheless, the mechanisms involved in these metabolic derangements remain obscure and warrant further studies. In the present investigation, we succeeded in achieving a reliable model of cellular lipid peroxidation and inflammation using Caco-2/15 cells and Fe/Asc oxygen radical-generating system that modified the activity of SOD2 and GPx enzymes. Scrutiny in the molecular mechanisms revealed the regulatory action of epigenetic mechanisms that functionally changed methylation of promoter CpG islands of *SOD* and *GPx* genes. The use of the Trolox and 5-AZA, as antioxidant and demethylating agents, respectively, confirmed the implication of lipid peroxidation and epigenetics processes since they alleviated MDA and methylation magnitude while limiting inflammatory extent.

To evaluate the influence of lipid peroxidation on the antioxidant defense in the current study, we used the Caco-2/15 cell line that spontaneously differentiates into polarized mature enterocytes, expresses several morphological and functional characteristics of the small intestine under standard culture conditions, and lends itself to the in *vitro* study of human gut in view of its efficient intestinal transport processes [25], [43]–[46]. Caco-2/15 cell monolayers have been, by far, the most utilized cell model to predict the flux of drugs across human small intestinal tissue [47], [48]. They have also been demonstrated to be extremely useful for the screening of antioxidant defense, OxS, inflammation and intestinal barrier as highlighted by various groups [49]–[51] and our own laboratory [25]–[27], [52]. The Caco-2/15 cells develop structural characteristics like those of a small intestinal enterocyte epithelium [53] and share functional similarities with enterocytes as a model to examine oral absorption [54], providing an alternative to the use of other intestinal models [55]. In order to induce robust lipid peroxidation, we used the complex of iron-ascorbate, since iron causes oxidative damage to biological macromolecules, alters the intracellular redox environment and is involved in numerous pathological states [25], [56]–[60], whereas ascorbic acid can amplify the oxidative potential of iron by promoting metal ion-induced lipid peroxidation [61]. It is noteworthy that the iron dose used in the current study is comparable with normal iron concentration in the gut [62]. We have extensively used iron/ascorbate as a strong oxygen-radical generating system in our previous studies. To provide only few examples, we could document the ability of these combined molecules to (i) initiate sturdy lipid peroxidation in native cell membranes thereby affecting regulatory enzymes [63]; (ii) disturb the assembly and secretion of lipoproteins, thereby resulting in abnormal lipid transport [61], [64]; (iii) provoke inflammatory reactions in intestinal cells [65]; (iv) induce various dysregulations [24]–[26], [66]; and (v) produce harmful effects on mitochondrial function and DNA integrity [27]. Before performing many of these studies, we examined the impact of Fe/Asc on lipid peroxidation as a function of concentration and incubation periods [65]. In addition, relevant studies from our laboratory demonstrated that a 6 h-period is sufficient to induce inflammation by Fe/Asc [26], [27], [52]. The deteriorations resulting from the exposure of Caco-2/15 cells to Fe/Asc are probably attributable to OxS, because the addition of the Trolox antioxidant simultaneously alleviated, without totally preventing, the occurrence of lipid peroxidation along with inflammation relief. Trolox was selected as an antioxidant because it represents a powerful agent inhibiting iron-mediated OxS and does not have any toxic effects on Caco-2/15 cell culture [67]. However, the incomplete neutralization of lipid peroxidation may be due to the insufficient efficiency of Trolox to scavenge the diversity of free radicals.

Since NF-κB is a redox-sensitive transcription factor that can be altered by OxS, we tested the expression of its subunits. We found that upon Fe/Asc -mediated lipid peroxidation, Caco-2/15 cells exhibited an activation of NF-κB in view of the increased expression of the p50/p65 heterodimer and decreased expression of IκB. In intestinal epithelial cells, the inflammatory response is largely controlled through regulation of the transcription nuclear factor NF-κB, which exists primarily in the cytosol as a p50/p65 heterodimer complexed with its inhibitor protein IκB. Activation of the IκB kinase complex by numerous stimuli leads to the phosphorylation of IκB, causing its dissociation from the NF-κB heterodimer and subsequent degradation by the proteasome. Loss of IκB reveals a nuclear localization sequence on the NF-κB heterodimer that allows its rapid translocation to the nucleus and facilitates transcription of pro-inflammatory proteins [68]. In our experiments, the pro-oxidant Fe/Asc system was capable of decreasing IκB protein expression directly in line with the hypothesis that the release and translocation of p50/p65 heterodimer to the nucleus take place, which may trigger the trans-activation of the inflammatory IL-6 gene. Apparently, Fe/Asc-mediated lipid peroxidation led to amplification of the inflammatory response in Caco-2/15 cells given the activation of COX-2 that catalyzes the formation of prostaglandins from arachidonic acid and is found in high levels in inflammatory state [69], [70].

We reasoned that excessive lipid peroxidation caused by Fe/Asc treatment in Caco-2/15 overwhelmed the antioxidant defense. Indeed, we noted activity stimulation of SOD2, the antioxidant defense that catalyzes the dismutation of the superoxide radical into hydrogen peroxide, which is generally converted to water by GPx. Our experiments showed an up-regulation of SOD2 activity along with a down-regulation of GPx while GR activity remained unchanged. Validation was obtained by RT-PCR that reveals the same trend of mRNA expression, suggesting an upstream transcriptional regulation. Of note, previous studies have reported that H_2_O_2_ accumulation could inhibit GPx [71], activate kinases capable of phosphorylating IKB, and promote the translocation of NF-κB components (i.e. p65, p50, p52) to the nucleus where they bind DNA promoters and trigger transcription [72]. In view of our findings related to mRNA elevation and available literature, one may posit that NF-κB activates SOD transcription [73]. GR activity remained unchanged probably because oxidized glutathione, which is the product generated by GPx, is reduced [74]. Our further studies illustrated the hypomethylation of the SOD2 promoter as well as the hypermethylation of the GPx2 promoter in Caco-2/15 cells exposed to Fe/Asc. Interestingly, we detected both hypomethylation and hypermethylation in our cellular model just as has been reported in tumors characterized by the paradoxical co-presence of local and global DNA hypomethylation together with the regional hypermethylayion of certain genes [75]. Similar situations were noted following short-term arsenic-exposed newborns, which resulted in a significant global hypomethylation and p53 hypermethylation [76]. Moreover, early overfeeding altered DNA methylation patterns of hypothalamic promoter regions: neuropeptide Y was methylated at low levels whereas the main anorexigenic neurohormone, proopiomelanocortin showed hypermethylation [77]. How this methylation imbalance evolves remains puzzling. It was hypothesized that chromatin structure changes occur during oncogenesis that predisposes to both demethylation and *de novo* methylation [78]. Additional studies are necessary to clarify this fascinating issue.

DNA methylation is controlled by DNA methyltransferase (DNMT), an enzyme that catalyzes the transfer of a methyl moiety from S-adenosyl-1-methionine to the 5-position of cytosines in the CpG dinucleotide [79]. In the present investigation, we employed the nucleotide analogue 5-AZA that inhibits the methylation of newly synthesis of DNA [80], [81], favors the proteasomal degradation of DNMT1 [82], and is associated with lower level of DNMT1 and DNMT3a protein expression [83]. The incubation of Caco-2/15 cells with 5-AZA led to the reduction, but not fully, of MDA levels which is enough to prevent NF-κB, COX-2, and IL-6 expressions. In addition, it increased IκB and reestablishing the enzymatic activity and gene expression of SOD2 and GPx2 by restoring DNA methylation. Our observations are in line with the studies reporting that 5-AZA had the capacity to normalize Keap1, a cytoplasmic anchor for nuclear factor E2-related factor 2, a transcription factor that induces most of the antioxidant enzymes [84]. Moreover, a number of reports indicated that 5-AZA altered DNA binding activation of NF-κB in macrophage [85], reversed inflammation (COX-2) in Epstein-Barr Virus-positive gastric epithelial cells [86] and inhibited IL-6 in a cohort of ulcerative colitis patients with varying degrees of dysplasia [87]. These observations can explain why Fe/Asc/5-AZA group does not generate inflammation, despite the high residual OxS.

In conclusion, our studies indicate that the Fe/Asc oxidant stress system leads to changes in SOD2 and GPx activities that are associated with epigenetic changes in promoter DNA methylation, and stimulation of inflammation markers. Further studies are needed to determine the role of this mechanism in different intestinal complex diseases, especially since important recent advances in epigenetics research have indicated that the loss of SOD2 activity itself may contribute to change in epigenetic regulation, establishing a vicious cycle driving further epigenetic instability [88].
