# Expression of Concern: Iron-Ascorbate-Mediated Lipid Peroxidation Causes Epigenetic Changes in the Antioxidant Defense in Intestinal Epithelial Cells: Impact on Inflammation

**DOI:** 10.1371/journal.pone.0267237

**Published:** 2022-04-14

**Authors:** The PLOS ONE Editors

Following the publication of this article [1], concerns were raised regarding results presented in Figs 3, 4 and 6. Specifically,

- The following results appear similar
  ○ The Fig 3D NF-κB panel and the Fig 3E IκB panel.
  ○ The Fig 3D and 3E β-actin panels.
  ○ Fig 6A GAPDH panel, Fig 6B GAPDH panel, Fig 6C GAPDH panel lanes 1–4, and Fig 6D GAPDH panel lanes 1–4. In addition, lanes 5 of the Fig 6C and 6D GAPDH panels also appear similar.
  ○ Fig 6B GPx panel lanes 2–4, and Fig 6D GPx panel lanes 1–3.
  ○ Fig 6C SOD2 panel lanes 1–2 and Fig 6D GPx panel lanes 4–5.
- When levels are adjusted to visualize background, there appear to be vertical irregularities suggestive of splice lines in the Fig 4B COX-2 panel, between lanes 1–2 and lanes 3–4.

The corresponding author stated that the similarities between reference blots (β-actin) are due to the stripping and re-probing of the same blot, and that some blot images were spliced during Fig preparation. However, this explanation does not clarify the concerns pertaining to the similarity between the GAPDH results presented in Fig 6A and 6B and Fig 6C and 6D, as these panels represent different experimental conditions, and in addition, these results present gel data which are unaffected by stripping and reprobing.

The corresponding author provided the image data presented in the S1 File to support the results presented in Figs 3D, 3E, 6B and 6D. The underlying data provided suggests that the wrong image was used for Fig 3E IκB. Assessment of the time stamp of the underlying data provided for Fig 6B GPx and respective GAPDH results suggests that these images were taken nearly 7 months apart. In addition, the underlying data for the Fig 6B and Fig 6D GAPDH results appear very similar, and assessment of the time stamp and file ID indicates that the images were taken in rapid succession. The underlying gel images provided for the Fig 6D GPx and respective GAPDH results match the published panels, but the image provided for the Fig 6B GPx panel does not match the published panel. The corresponding author also provided the underlying data for the graphs presented in Fig 6C and 6D, which have been provided in the S2 File.

The corresponding author stated that the underlying data for other Fig panels in question were provided to *PLOS ONE* in 2014 but are no longer available in the laboratory records. PLOS is unable to access the journal’s 2014 correspondence records for this case. We sincerely regret that this case was not resolved much sooner after the prior correspondence.

In the absence of supporting image data to confirm the image results presented in Figs 4B, 6A, and 6C, and the underlying individual level data to support the results presented in the graphs of Figs 3, 4, 6A, and 6B, we cannot clarify whether the reported claims based on these Figs are reliable. Given the concerns regarding the preparation of the data presented in Figs 3, 4, and 6, the *PLOS ONE* Editors issue this Expression of Concern to notify readers and relay the supporting data provided by the corresponding author.

## Supporting information

S1 File Underlying image data for results presented in Figs 3D, 3E, 6B and 6D. (PPT)

S2 File Individual level data for results presented in Fig 6C and 6D. Each value is the percentage calculated from the densitometric ratio of gene of interest/GAPDH (using UN-SCAN-IT gel 6.1) in different test conditions normalized with the mean of the ratio of gene of interest to GAPDH in the control condition software using GraphPad Prism 5.01 Software. The graph represents MEANS +/- SEM, and the outlier values (data that exceed +/- 2 standard deviations, highlighted in blue in the S2 File) were excluded from the graph.
(XLSX)
