# Supplementary material for: Expression of Concern: Iron-Ascorbate-Mediated Lipid Peroxidation Causes Epigenetic Changes in the Antioxidant Defense in Intestinal Epithelial Cells: Impact on Inflammation
Source: PLoS One. 2022 Apr 14;17(4):e0267237. doi: 10.1371/journal.pone.0267237 (PMC9009708; doi:10.1371/journal.pone.0267237)
Supplement: S1 File — (PPT) [file pone.0267237.s001.ppt]

## Slide 1
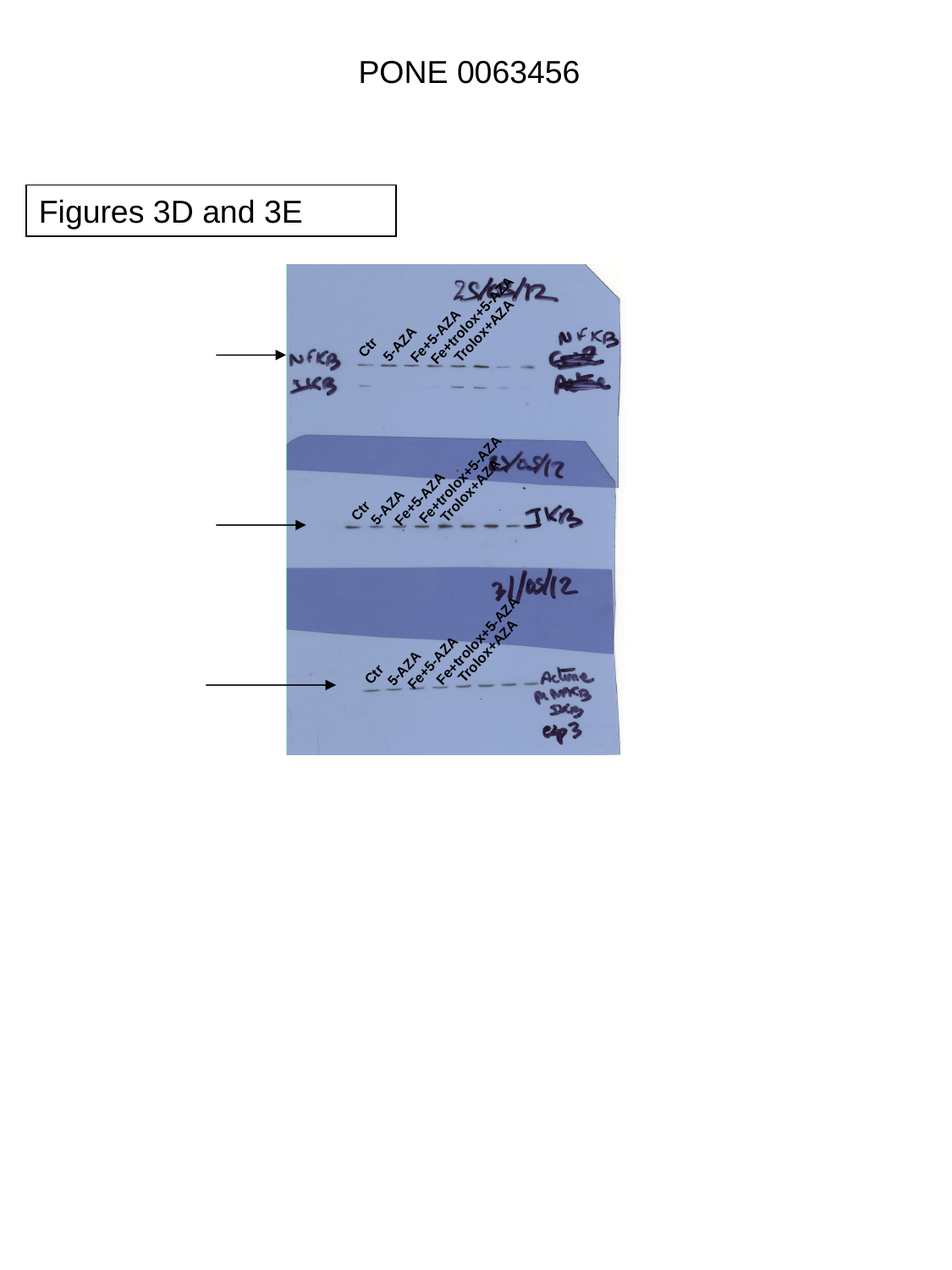

PONE 0063456
Figures 3D and 3E
Fe+trolox+5-AZA
Trolox+AZA
Fe+5-AZA
5-AZA
Ctr
Fe+trolox+5-AZA
Trolox+AZA
Fe+5-AZA
5-AZA
Ctr
Fe+trolox+5-AZA
Trolox+AZA
5-AZA
Fe+5-AZA
Ctr

## Slide 2
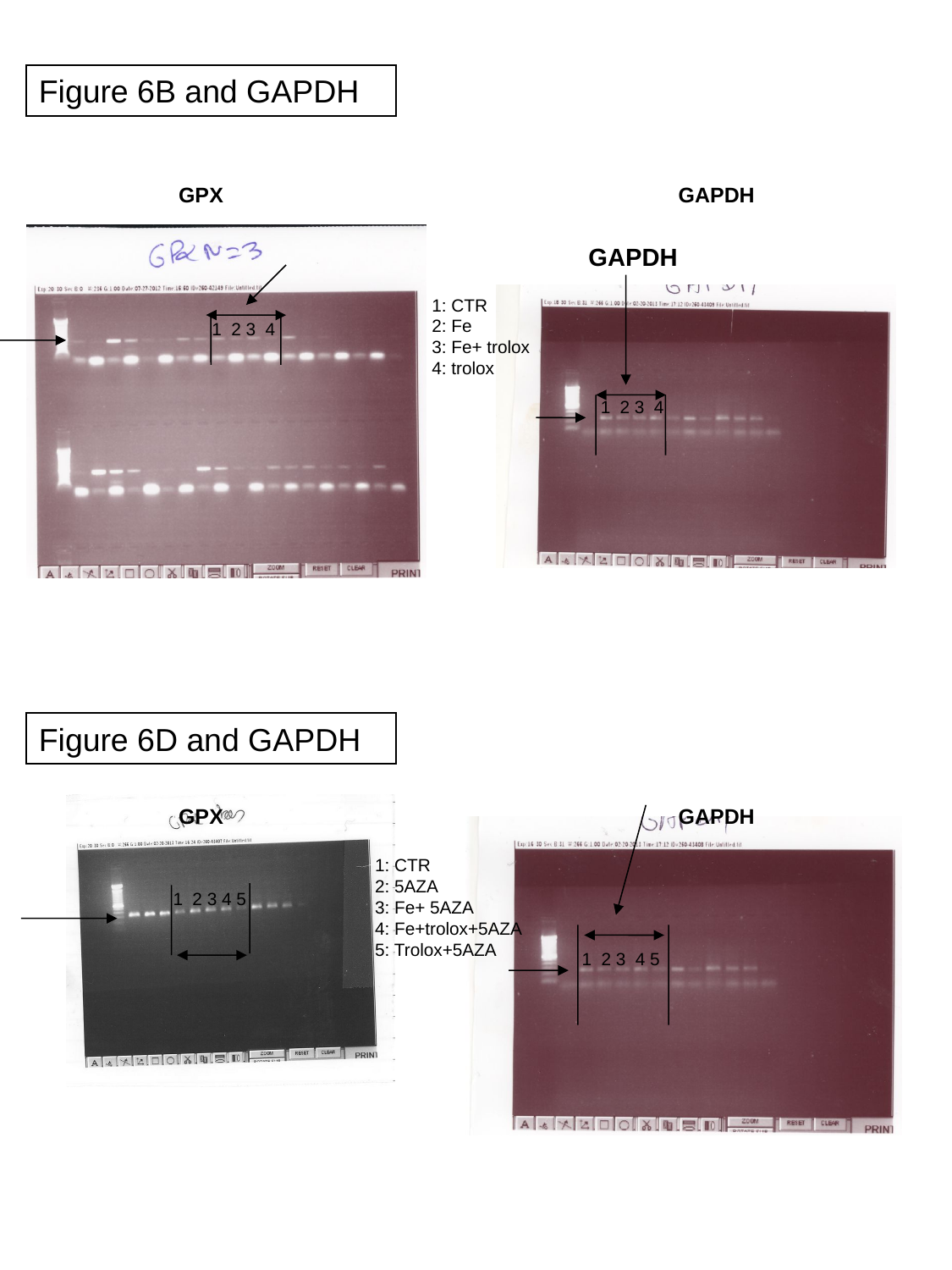

Figure 6B and GAPDH
GPX
GAPDH
GAPDH
1: CTR
2: Fe
3: Fe+ trolox
4: trolox
1 2 3 4
1 2 3 4
Figure 6D and GAPDH
GPX
GAPDH
1: CTR
2: 5AZA
3: Fe+ 5AZA
4: Fe+trolox+5AZA
5: Trolox+5AZA
1 2 3 4 5
1 2 3 4 5
